# Targeting FLT3 Signaling in Childhood Acute Myeloid Leukemia

**DOI:** 10.3389/fped.2017.00248

**Published:** 2017-11-20

**Authors:** Amy N. Sexauer, Sarah K. Tasian

**Affiliations:** ^1^Dana-Farber Cancer Institute, Boston, MA, United States; ^2^Boston Children’s Hospital, Department of Pediatrics, Division of Pediatric Hematology/Oncology/Stem Cell Transplant, Boston, MA, United States; ^3^Children’s Hospital of Philadelphia, Division of Oncology, Center for Childhood Cancer Research, Philadelphia, PA, United States; ^4^Perelman School of Medicine, University of Pennsylvania, Philadelphia, PA, United States

**Keywords:** acute myeloid leukemia, clinical trials, FLT3, kinase inhibitor, pediatrics

## Abstract

Acute myeloid leukemia (AML) is the second most common leukemia of childhood and is associated with high rates of chemotherapy resistance and relapse. Clinical outcomes for children with AML treated with maximally intensive multi-agent chemotherapy lag far behind those of children with the more common acute lymphoblastic leukemia, demonstrating continued need for new therapeutic approaches to decrease relapse risk and improve long-term survival. Mutations in the FMS-like tyrosine kinase-3 receptor gene (*FLT3*) occur in approximately 25% of children and adults with AML and are associated with particularly poor prognoses. Identification and development of targeted FLT3 inhibitors represents a major precision medicine paradigm shift in the treatment of patients with AML. While further development of many first-generation FLT3 inhibitors was hampered by limited potency and significant toxicity due to effects upon other kinases, the more selective second- and third-generation FLT3 inhibitors have demonstrated excellent tolerability and remarkable efficacy in the relapsed/refractory and now *de novo FLT3*-mutated AML settings. While these newest and most promising inhibitors have largely been studied in the adult population, pediatric investigation of FLT3 inhibitors with chemotherapy is relatively recently ongoing or planned. Successful development of FLT3 inhibitor-based therapies will be essential to improve outcomes in children with this high-risk subtype of AML.

## Introduction

Acute myeloid leukemia (AML) is a group of biologically heterogeneous diseases that comprise 20% of pediatric and 80% of adult acute leukemias (1, 2). It is estimated that 21,380 people in the United States will be diagnosed with AML in 2017, and 10,590 of these patients will die from leukemia (3). While outcomes for children with *de novo* AML have improved over the past several decades, event-free survival (EFS) and overall survival (OS) remain suboptimal at approximately 60 and 70%, respectively (4). Relapsed disease and poor response to salvage therapy remain significant hurdles in achieving cure.

**Figure 1:**
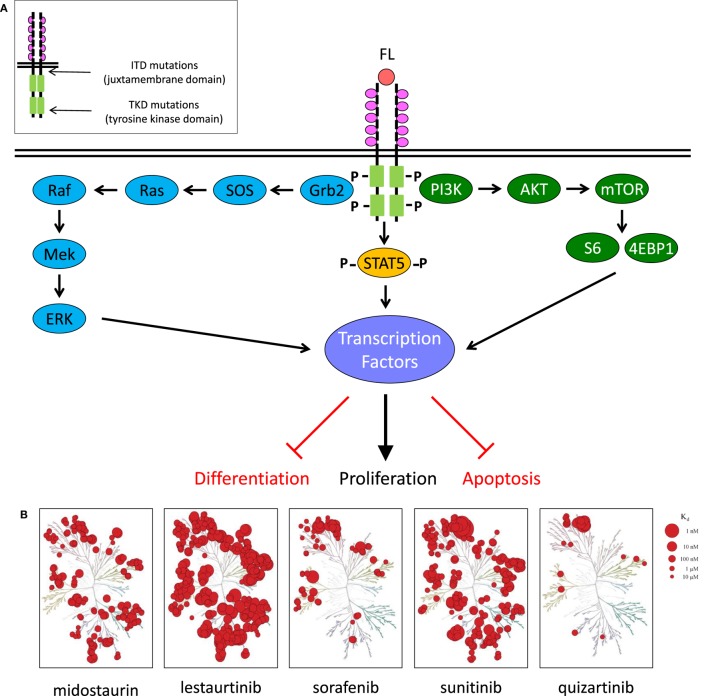
FLT3 signaling in acute myeloid leukemia and clinically relevant FLT3 tyrosine kinase inhibitors. **(A)** FLT3 receptor and downstream signaling targets schema. **(B)** KinomeScan dendrograms (http://lincs.hms.harvard.edu/kinomescan/) demonstrating relative potency and selectivity of FLT3 inhibitors [adapted from Zarrinkar et al. (12); used with permission]. Interactions with *K*_d_ <3 µM are shown. Red circles designate kinases bound. Circle size specifies relative binding affinity. Dendrogram data are not available for ponatinib, crenolanib, or gilteritinib.

FMS-like tyrosine kinase-3 (FLT3; CD135) is a 993 amino acid single transmembrane type III receptor tyrosine kinase in the same family as the structurally similar stem cell growth factor receptor c-KIT (CD117), colony-stimulating factor-1 receptor (CSF1R; CD115), and platelet-derived growth factor receptor (PDGFR) (5–8). FLT3 has a single extracellular ligand-binding domain with five immunoglobulin-like folds, a juxtamembrane domain, and a single cytoplasmic tyrosine kinase domain separated by a kinase insert region (Figure 1A). FLT3 signaling plays a critical role in hematopoiesis and is expressed on CD34+ hematopoietic stem/progenitor cells, but its surface expression is lost during cellular differentiation (7, 9, 10). Normally, the FLT3 receptor is stimulated by FLT3 ligand, leading to receptor dimerization with subsequent activation of its tyrosine kinase domain, autophosphorylation, and binding of SH2 domain-containing proteins. Activated FLT3 then phosphorylates downstream targets, including STAT5, SHIP, and SHP-2, and signals through critical oncogenic pathways such as Ras/Raf/MAPK and PI3K/Akt/mTOR (5, 6, 11) (Figure 1B).

Wild-type *FLT3* is overexpressed in most cases of B-lymphoblastic leukemia and AML and in a smaller percentage of T-lineage ALL and chronic myeloid leukemia (CML) in blast crisis (13). Mutations in *FLT3* are one of the most common genetic alterations in AML and are associated with high rates of relapse in adults and children (14–16). Activating *FLT3* mutations are classified into two types: (1) internal tandem duplication (FLT3–ITD) mutations, which are 3–400 bp in-frame duplications located in the juxtamembrane domain and (2) activating point mutations, which are found in the tyrosine kinase domain (FLT3–TKD) and most often involve residue D835 (17–19). ITD and TKD mutations occur in approximately 25 and 10% of adult AML cases, respectively (20, 21). Recent studies have reported similar incidence of ITD and activating TKD mutations in childhood AML (15, 22–25). Numerous clinical trials have demonstrated inferior clinical outcomes in patients with FLT3–ITD AML (14–16, 26, 27).

Adults with newly diagnosed AML are generally treated with cytarabine- and anthracycline-based induction chemotherapy followed by consolidation therapy. Allocation to subsequent hematopoietic stem cell transplant (HSCT) is usually based on cytogenetic risk stratification and transplant eligibility status (2). With this approach, 5-year OS for adults with AML is approximately 40%. However, several studies have demonstrated significantly decreased duration of first remission (CR1) and 5-year OS of approximately 15% in adults with FLT3–ITD AML versus those without *FLT3* mutations (28–32). Children with FLT3–ITD AML treated on Children’s Cancer Group and Pediatric Oncology Group trials fared similarly poorly with 30% 4-year EFS when treated with conventional multi-agent chemotherapy (15). Higher mutant-to-wild-type FLT3 allelic ratios have also been associated with increasingly inferior outcomes in children treated on Dutch Children’s Oncology Group and Children’s Oncology Group (COG) studies (15, 33). In a recent subgroup analysis, the COG phase 3 trial AAML0531 reported decreased relapse rates in children with FLT3–ITD AML with addition of the CD33-targeting antibody-drug conjugate gemtuzumab ozogamicin to standard chemotherapy (16, 34), demonstrating potential for improved clinical outcomes in this high-risk patient population with inclusion of targeted therapies.

Given the significant negative prognostic effects of FLT3–ITD mutations in AML and the relative frequency of these alterations, therapeutic targeting of aberrant FLT3 signaling has been a major research focus with goals of decreasing relapse and improving survival. Tyrosine kinase inhibitors (TKIs) are small molecules that inhibit the enzymatic activity of tyrosine kinases and block downstream signaling activation. Treatment of adults with CML with the SRC/ABL inhibitor imatinib is one of the major early successes of modern precision medicine (35, 36). Imatinib targets the oncogenic BCR–ABL fusion protein resulting from t(9;22) (Philadelphia chromosome; Ph+) by inhibiting the active site of the ABL1 kinase. Treatment of patients with CML and Ph+ ALL with imatinib or related TKIs markedly improved remission rates and long-term survival versus prior interferon and chemotherapy and is now considered standard-of-care therapy (37–40). Similarly, FLT3 inhibitor treatment of patients with *FLT3*-mutated AML has been investigated for the past decade with promising results of several studies recently reported. This review discusses the current landscape of and future potential for clinical testing of FLT3 inhibitors in adults and children with *FLT3*-mutated AML (Table 1).

**Table 1:** Current clinical trials of FLT3 inhibitors in children and adults with AML.

Drug	Clinical trial number (pediatric)	Clinical trial number (adult)	Phase of testing	Reference
Midostaurin	NCT00866281^a^ (ITCC-024, CPKC412A2114)	NCT00045942	1, 2 (with HSCT)	Zwaan et al. (41)^a^ Stone et al. (42)^b^ Stone et al. (43)^c^
		NCT00651261^b,c^		
		NCT01093573		
		NCT01477606		
		NCT01830361		
		NCT01846624		
		NCT01883362		
		NCT02634827		

Lestaurtinib	NCT00469859 (COG AAML06P1)	NCT00030186	1, 2	Levis et al. (44)^d^
		NCT00079482^d^		

Sorafenib	NCT00908167^e^	NCT00373373	1, 2, 3 (with HSCT)	Inaba et al. (45)^e^ Widemann et al. (46)^f^ Rollig et al. (47)^g^ Chen et al. (48)^h^ Ohanian et al. (49)^i^
	NCT01371981 (COG AAML1031)	NCT00542971		
	NCT01445080^f^ (COG ADVL0413)	NCT00893373^g^		
		NCT01398501^h^		
		NCT02156297		
		NCT02196857^i^		
		NCT02867891		

Sunitinib	None	NCT00783653^j^	1,2	Fiedler et al. (50)^j^
				Fiedler et al. (51)

Quizartinib	NCT01411267^k^ (TACL T2009-004)	NCT00462761^l^	1, 2, 3	Cooper et al. (52)^k^ Cortes et al. (53)^l^ Perl et al. (54)^m^ Schiller et al. (55)^n^
		NCT00989261^m^		
		NCT01390337		
		NCT01468467		
		NCT01565668^n^		
		NCT02668653		
		NCT02984995		

Ponatinib	None	NCT00660920^o^	1, 2	Cortes et al. (56)^o^
		NCT02428543		

Crenolanib	NCT02270788 (SJCRH RELHEM2)	NCT01522469^p^	1, 2, 3 (with HSCT)	Cortes et al. (57)^p^ Galanis et al. (58)^q^
		NCT01657682^q^		
		NCT02283177		
		NCT02298166		
		NCT02400255		
		NCT02400281		

Gilteritinib	None	NCT02014558^r,s^	1, 2, 3 (with HSCT)	Perl et al. (59)^r^ Altman et al. (60)^s^ Cortes et al. (61)^t^
		NCT02421939		
		NCT02752035^t^		
		NCT02927262		
		NCT02997202		
		NCT03070093		

*COG, Children’s Oncology Group; HSCT, hematopoietic stem cell transplantation; ITCC, Innovative Treatments for Childhood Cancer European consortium; NCT, clinicaltrials.gov trial number; SJCRH, St. Jude Children’s Research Hospital; TACL, Therapeutic Advances in Childhood Leukemia and Lymphoma consortium*.

*Corresponding clinical trial publications or abstracts are annotated with superscripted characters for each inhibitor where available*.

## Current FLT3 TKIs IN Clinical Use

### Midostaurin

Midostaurin (PKC412) is a first-generation oral FLT3 inhibitor initially named due to its inhibitory effects upon protein kinase C. Midostaurin was subsequently recognized as a promiscuous kinase inhibitor with strong inhibitory effects also against the vascular endothelial growth factor receptor (VEGFR), PDGFRα and β, spleen tyrosine kinase (SYK), c-KIT, and FLT3 (62). Midostaurin has been studied extensively in adults with relapsed/refractory AML. Initially, midostaurin monotherapy was observed to induce a “blast response” (≥50% reduction in blast counts in peripheral blood and/or bone marrow) in 70% of adults with FLT3–ITD AML and in 30–40% with *FLT3*-wild-type AML (62, 63). However, achievement of long-term remission was rare (63). Subsequent trials, thus, combined midostaurin with induction chemotherapy and reported improved CR rates in patients with FLT3–ITD AML (42). These studies were followed by the Cancer and Leukemia Group B 10603 RATIFY trial, an international double-blind randomized controlled study comparing standard chemotherapy without or with midostaurin in adults (18–59 years) with *de novo* FLT3–ITD or FLT3–TKD AML. Addition of midostaurin to chemotherapy significantly improved median EFS (8.2 versus 3.0 months) and OS (74.7 versus 25.6 months) compared to patients treated with chemotherapy and placebo (43). The precise impact of HSCT and potential differential responses of midostaurin treatment between FLT3–ITD and TKD patients is not fully known (43, 64). Based on results of this trial, midostaurin was recently approved by the United States Food and Drug Administration (FDA) for use in adults with *de novo FLT3*-mutated AML (64).

To date, one trial of midostaurin has been conducted in children with leukemia (41). An Innovative Therapies for Children with Cancer European consortium-led phase 1/2 dose escalation study aimed to establish the safety, tolerability, and efficacy of midostaurin in children and adolescents 3 months–18 years of age with either relapsed/refractory *FLT3*-mutated AML or *KMT2A*-rearranged ALL (which overexpresses wild-type FLT3). The trial closed early due to inadequate accrual. While the number of subjects studied was too small for more rigorous analysis, 5 of 15 patients with AML and 3 of 13 patients with *KMT2A*-rearranged ALL had partial or complete responses with midostaurin monotherapy with OS of 3.7 (AML) and 1.4 months (ALL), respectively (41).

### Lestaurtinib

Lestaurtinib (CEP-701) is another first-generation multi-kinase inhibitor with activity against FLT3, Janus kinase 2, and tropomyosin receptor kinase A. One randomized trial tested lestaurtinib administration after induction chemotherapy in adult patients with AML in first relapse and demonstrated no survival benefit with lestaurtinib addition versus chemotherapy only (44). However, pharmacodynamic assessment of *in vivo* signaling inhibition by plasma inhibitory activity (PIA) assays demonstrated that few patients achieved sustained FLT3 inhibition (65), limiting conclusions about the potential efficacy of lestaurtinib in this population (44, 66). The UK AML15 and AML17 trials also studied lestaurtinib addition versus standard chemotherapy in 500 adults with AML harboring FLT3-activating mutations and showed no significant improvement in OS (67). However, PIA assays conducted in this trial correlated with significantly decreased relapse rates in lestaurtinib-treated patients who consistently achieved >85% FLT3 inhibition, further corroborating the importance of pharmacodynamic correlation (67).

The COG conducted an analogous pilot trial AAML06P1 (NCT00469859) in children and adolescents and young adults (AYAs) <30 years of age with relapsed/refractory AML. These patients were induced with cytarabine and idarubicin, then treated with lestaurtinib. The trial closed after the safety phase demonstrated tolerable combination dosing, but without completion of efficacy phase testing (66). In the pediatric population, lestaurtinib has been better studied in infants with wild-type *FLT3*-overexpressing *KMT2A*-rearranged ALL. Despite very promising preclinical data, the randomized COG phase 3 trial AALL0631 (NCT00557193) failed to demonstrate benefit of lestaurtinib addition to chemotherapy in infants with *KMT2A*-rearranged ALL, although achievement of sustained FLT3 inhibition as measured by PIA assays was variable among patients and across therapy phases (68).

### Sorafenib

Sorafenib (BAY 43-9006) is another first-generation pan-kinase inhibitor with activity against Raf, c-KIT, PDGFR, VEGFR, and FLT3 (69). Sorafenib is FDA-approved for the treatment of adults with renal cell, hepatocellular, and thyroid carcinomas (70–72). Initial studies of sorafenib monotherapy in adults with *FLT3*-mutated AML demonstrated safety and tolerability with minimal toxicity (69, 73). Subsequent trials investigated the safety and efficacy of combining sorafenib with chemotherapy in adults with *de novo* AML. One phase 2 study at the MD Anderson Cancer Center tested sorafenib with cytarabine and idarubicin in 62 newly diagnosed patients, 19 of whom had FLT3–ITD AML. While response rates were higher in patients with *FLT3* mutations, no differences in EFS or OS were observed (74). The successor international phase 2 SORAML trial randomized 267 adults (ages 18–60 years) to induction chemotherapy with cytarabine and daunomycin, followed by high-dose cytarabine consolidation therapy with sorafenib or placebo. Intermediate-risk patients with sibling donors and high-risk patients with any matched donor in first remission were allocated to subsequent allogeneic HSCT. The SORAML trial demonstrated clear benefit in the sorafenib-treated cohort with respect to relapse-free survival (21 months median EFS versus 9 months for placebo-treated patient), although did not improve OS rates (47).

A phase 1 trial conducted at St. Jude Children’s Research Hospital (SJCRH) first studied sorafenib monotherapy in children with relapsed/refractory AML, then in combination with clofarabine and cytarabine. Five of 12 enrolled patients had FLT3–ITD AML. Responses were observed in most patients, including all five FLT3–ITD patients, with complete remission (CR) in four patients, CR with incomplete count recovery (CRi) in two patients, and a partial response in one patient (45). A concomitant COG phase 1 study of sorafenib in children with relapsed/refractory solid tumors or leukemias also identified tolerable dosing in children and reported complete responses in two of eight patients with FLT3–ITD AML, enabling subsequent HSCT (46). The COG phase 3 trial AAML1031 (NCT01371981) is currently assessing the efficacy of non-randomized sorafenib addition to chemotherapy and best available donor HSCT for children and AYAs with *de novo* FLT3–ITD AML. The study was amended to include a 1-year sorafenib maintenance phase post-HSCT based upon smaller studies reporting potential efficacy of this post-transplant strategy to minimize relapse risk (75). Results from the AAML1031 sorafenib arm are not yet available, although some dosing modifications have been required due to incidence of hand–foot syndrome, hypertension, and cardiac dysfunction (Children’s Oncology Group Myeloid Diseases Committee, unpublished).

### Sunitinib

Sunitinib (SU11248), another multi-kinase inhibitor with activity against FLT3, has been studied in adult patients with *FLT3*-mutated AML with reported similar efficacy as sorafenib (50). Tolerability and preliminary efficacy of sunitinib was assessed in children with relapsed/refractory solid tumors via the COG trials ADVL0413 and ACNS1021 (76, 77). Sunitinib has been studied in a small number of children with FLT3–ITD AML who failed prior sorafenib treatment (78).

### Quizartinib

Given the potential for increased toxicities of the first-generation FLT3 inhibitors secondary to effects upon other kinases and/or poor pharmacodynamic properties, second-generation inhibitors with greater anti-FLT3 potency were developed. Quizartinib (AC220) is the first drug specifically designed as a FLT3 inhibitor and has 10–50 times greater *in vivo* potency than first-generation inhibitors (12, 79, 80). Quizartinib also has moderate activity against c-KIT (81). Initial phase 1 studies of quizartinib monotherapy in adults with relapsed/refractory AML demonstrated tolerability and preliminary efficacy (53), and subsequent phase 2 trials have reported high response rates in both younger and older adults with relapsed/refractory AML (54, 82). Other phase 2 and phase 3 studies are currently investigating the efficacy of quizartinib in combination with chemotherapy. Preclinical and clinical studies have now demonstrated resistance mutations in patients with FLT3–ITD AML treated with quizartinib, particularly the F691L gatekeeper and D835/I836 activation loop mutations (83, 84).

The Therapeutic Advances in Childhood Leukemia/Lymphoma (TACL) consortium conducted a phase 1 trial of quizartinib with cytarabine and etoposide in 17 children (ages 1 month to 21 years) with relapsed/refractory AML or *KMT2A*-R ALL. Quizartinib was well-tolerated without dose-limiting toxicity, and correlative PIA assays demonstrated near-complete pharmacodynamic inhibition of FLT3 at all tested doses (52). All seven patients with FLT3–ITD AML had marked reduction in medullary leukemia burden with three patients achieving CR or CRi and proceeding to allogeneic HSCT. Similar responses were not observed in children with wild-type FLT3 AML or *KMT2A*-rearranged ALL (52). These data further support potential improved anti-leukemic activity of more selective FLT3 inhibitors.

### Ponatinib

Ponatinib (AP24534) is a third-generation multi-kinase inhibitor with activity against BCR–ABL and FLT3. Ponatinib is currently FDA-approved for treatment of adults with TKI-resistant CML or Ph+ ALL (85). In preclinical studies, ponatinib had significant anti-leukemia activity against AML specimens with FLT3–ITD or TKD mutations, including the F691I gatekeeper (86–88). A current phase 1/2 trial is studying the safety and efficacy of ponatinib in combination with cytarabine in adults with FLT3–ITD AML (NCT02428543). Ponatinib currently has an FDA black box warning regarding serious risk of arterial thrombosis and hepatotoxicity (89). No formal studies of ponatinib in children have been conducted, although anecdotal cases of compassionate usage in pediatrics have been reported (90).

### Crenolanib

Crenolanib (CP-868596) was originally designed as a PDGFR inhibitor, although later studies also demonstrated its potency as a FLT3 inhibitor. Due to its short half-life, crenolanib requires thrice-daily dosing. Early data suggest that this third-generation TKI has robust activity against both FLT3–ITD and FLT3–TKD mutations, including those that confer resistance to quizartinib (58, 91, 92). Crenolanib monotherapy has been studied in several early-phase trials in adults with relapsed/refractory *FLT3*-mutated AML with encouraging activity (93), and combination trials in patients with newly diagnosed FLT3–ITD AML have demonstrated promising results (94).

Tolerable pediatric dosing of crenolanib monotherapy was established *via* a phase 1 trial conducted at SJCRH in children with central nervous system gliomas, which have activated PDGFR signaling (95). The current SJCRH RELHEM2 phase 1 trial (NCT02270788) is assessing the safety of combined crenolanib and sorafenib in children with relapsed or refractory hematologic malignancies.

### Gilteritinib

Gilteritinib (ASP2215), the newest third-generation oral FLT3 inhibitor, is the most potent and selective FLT3 inhibitor developed to date with moderate additional activity against the AXL kinase. In preclinical studies, gilteritinib has *in vitro* anti-FLT3–ITD activity that equals or surpasses that of other FLT3 inhibitors previously discussed. Gilteritinib is also active against FLT3–TKD resistance mutations and does not appreciably inhibit c-KIT (96), thereby potentially avoiding the myelosuppressive effects of quizartinib that have been observed in clinical trials (97). A first-in-human phase 1/2 trial evaluated gilteritinib monotherapy in adults with relapsed/refractory AML. This study reported excellent tolerability of gilteritinib and a 30% CR/CRi rate in heavily pretreated patients with many patients achieving deep molecular remission (59, 60). Gilteritinib is now under investigation in adults with relapsed and refractory *FLT3*-mutated AML via a randomized double-blinded phase 3 registration trial (NCT02997202) (Table 1). The FDA also recently granted orphan drug designation for gilteritinib for patients with *FLT3*-mutated AML. A pediatric development program for gilteritinib is planned.

## Future Directions

Despite maximal therapeutic intensification and significant improvements in supportive care, more than one-third of children with AML still die from leukemia or associated complications. *FLT3*-mutated AML is a particularly high-risk leukemia subtype in both adults and children, and the potential for selective FLT3 inhibitors to decrease relapse risk and improve cure rates is alluring. Initial trials of first-generation FLT3 inhibitors have validated FLT3 as a viable therapeutic target in AML and expedited FDA approval of midostaurin for adults with *FLT3*-mutated AML is a major recent achievement (64). However, poor pharmacokinetic properties or unfavorable toxicities of many multi-kinase inhibitors have limited usage in some patients. Trials of more selective second- and third-generation FLT3 inhibitors in adults with relapsed/refractory AML have established safety and tolerability of TKI monotherapy and in combination with chemotherapy, as well as exciting potential efficacy (14).

It is probable, perhaps certain, that children with FLT3 signaling-driven AML will similarly benefit from addition of FLT3 inhibition to chemotherapy. The newest and more selective inhibitors, quizartinib, crenolanib, and gilteritinib, have demonstrated very promising activity in adults with relapsed/refractory and newly diagnosed AML, but have been minimally or not studied to date in children. These agents merit broader clinical investigation in pediatrics. Emerging data from sorafenib maintenance studies also demonstrate the potential importance of such therapeutic strategies in the post-HSCT setting (75, 98). In addition, preclinical studies of FLT3-targeting chimeric antigen receptor T cell immunotherapy have demonstrated potent anti-leukemia activity in cell line (99, 100) and patient-derived xenograft models (Tasian, unpublished), further validating FLT3 as a robust therapeutic target in childhood AML.

Acquisition of resistance mutations following FLT3 inhibitor therapy remains a major source of treatment failure, although the incidence of such mutations in children with AML is not fully known. It is plausible that combining FLT3 inhibitors with chemotherapy may decrease the incidence of resistance mutations that occur with inhibitor monotherapy, analogous to lower mutation rates often observed in children with Ph+ ALL treated with TKI and chemotherapy (101, 102). However, major challenges exist in the study of new drugs in the pediatric population, including the relative rarity and genetic heterogeneity of childhood AML, rapid disease progression which may hamper trial enrollment, and the ability to partner with pharmaceutical companies to access novel agents for study in young children. Nonetheless, prospective clinical evaluation of exciting next-generation FLT3 inhibitors specifically in children with *FLT3*-mutated AML is ongoing or on the imminent horizon. Such important clinical investigation is critical to improve remission and decrease relapse in this highest-risk population of children with AML, potentially also reducing the significant toxicities associated with salvage therapy.

## Author Contributions

ANS and SKT wrote and edited the manuscript. Both authors approved the final version.

## Conflict of Interest Statement

The authors declare that the research was conducted in the absence of any commercial or financial relationships that could be construed as a potential conflict of interest.
